# Visomitin as a differentiation-inducing therapeutic agent through SYK inhibition in AML

**DOI:** 10.3389/fphar.2026.1741351

**Published:** 2026-02-24

**Authors:** Byeol-Eun Jeon, Chan-Seong Kwon, Ji-Eun Lee, Su-Ji Lee, Youngseuk Cho, Ho-Jin Shin, Sang-Woo Kim, Youngmi Jung

**Affiliations:** 1 Department of Integrated Biological Science, College of Natural Sciences, Pusan National University, Busan, Republic of Korea; 2 Department of Statistics, Pusan National University, Pusan, Republic of Korea; 3 Division of Hematology-Oncology, Department of Internal Medicine, Biochemical Research Institution, Pusan National University Hospital, Pusan National University School of Medicine, Busan, Republic of Korea; 4 Department of Biological Sciences, College of Natural Sciences, Pusan National University, Busan, Republic of Korea

**Keywords:** acute myeloid leukemia, apoptosis, differentiation, drug repositioning, reactive oxygen species, spleen associated tyrosine kinase

## Abstract

**Background:**

Acute myeloid leukemia (AML) is an aggressive hematological malignancy characterized by the rapid proliferation of immature myeloblasts and resistance to apoptosis. Overcoming the differentiation block and apoptotic resistance remains a major challenge in AML therapy. Visomitin, a mitochondria-targeted antioxidant, has shown protective effects in other contexts, but its potential in AML has not been explored.

**Methods:**

We examined the effects of Visomitin on AML cell differentiation and apoptosis using flowcytometry, including CD11b, CD14 staining and ROS measurement. Western blot analysis of Bcl-2 family proteins and p21/p16/Rb axis. Potential underlying mechanisms were explored through SYK activation. Additionally, primary AML patient samples were tested to assess translational relevance, and *in vivo* efficacy was evaluated in a xenograft mouse model.

**Results:**

Treatment with Visomitin promoted differentiation of AML cells, as indicated by increased CD14 expression, and induced apoptosis by downregulating anti-apoptotic proteins (Mcl-1, Bcl-XL) while upregulating pro-apoptotic factors (Bak, Bax). Mechanistic studies suggested that Visomitin-induced ROS accumulation enhances AML differentiation and apoptosis. Notably, Visomitin selectively increased ROS in AML cells while reducing ROS levels in normal myeloid cells. Pharmacological and genetic rescue experiments further imply that Visomitin’s anti-AML effects are mediated by ROS-dependent inhibition of SYK. *In vivo*, Visomitin suppressed tumor growth and elevated ROS within tumors. Furthermore, *ex vivo* treatment of primary AML cells reduced proliferation, highlighting potential clinical applicability.

**Conclusion:**

These findings suggest that Visomitin exerts potent anti-leukemic effects by simultaneously promoting differentiation and apoptosis through ROS-mediated SYK inhibition. The selective activity against malignant cells and favorable *in vivo* efficacy suggest that Visomitin is a potential therapeutic agent for AML.

## 1 Introduction

Acute myeloid leukemia (AML) is a malignant tumor characterized by arrested differentiation of hematopoietic progenitor cells and abnormal proliferation of cancer cells (Tenen, 2003; Nowak et al., 2009). Acute promyelocytic leukemia (APL), one of aggressive subtypes of AML, has shown dramatically improved outcomes by a combination of all-trans retinoic acid (ATRA) and arsenic trioxide (ATO). It turned out that ATRA/ATO treatment targeted differentiation blockade, which efficiently induced differentiation of APL cells and led to cell cycle arrest and apoptosis (Niu et al., 1999; de Thé, 2018), suggesting that differentiation-inducing therapy may hold great promise as a treatment for AML.

Despite the remarkable success of differentiation therapy in APL, it also showed clear limitations in other subtypes of AML, necessitating novel therapeutic agents based on better understanding of the disease. Over the past decades, however, the pace of new drug approvals has been slow, and the traditional drug development process has been increasingly lengthy and expensive. Thus, drug repositioning (DR), identification of different indications for existing drugs, has recently gained considerable attention as new strategies for drug discovery and development are needed. Especially in oncology, DR has emerged as a therapeutic innovation because it can be rapidly advanced to clinical trials based on previously published and readily available data (Hurle et al., 2013).

In this study, to fill this clinical need, we used flow cytometry analysis for phenotype-based screening of chemical library to identify compounds with the ability to induce differentiation in AML cells. Visomitin (also known as SKQ1) was selected for further preclinical evaluation as it was a compound that significantly induced differentiation and apoptosis in AML.

Visomitin, also known as SkQ1, is a novel mitochondria-targeted antioxidant composed of a plastoquinone moiety linked to a decyltriphenylphosphonium (TPP^+^) cationic tail (Dai et al., 2017; Huang et al., 2024). This unique structure enables SkQ1 to selectively accumulate within mitochondria at concentrations up to a thousand times higher than that of its uncharged counterpart, plastoquinone, thereby exerting potent antioxidant activity (Skulachev, 2012; Bazhin et al., 2016). Based on its strong efficacy against oxidative stress, Visomitin eye drops were developed by V. P. Skulachev. and have been used in the treatment of ocular surface diseases such as dry eye disease and corneal injury (Petrov et al., 2016). Moreover, recent studies have suggested that Visomitin may also exhibit therapeutic effects in cardiovascular diseases (Yang et al., 2022). Despite its known applications in inflammatory disorders, aging, and wound healing processes (Demianenko et al., 2010; Dai et al., 2014; Chelombitko et al., 2017), the potential role of Visomitin as an anti-cancer agent has not yet been fully explored. Here, we determined visomitin’s anti-leukemic activities using multiple AML cell lines and murine xenograft models, and AML patient samples.

## 2 Materials and methods

### 2.1 Reagents and antibodies

The FDA-Approved and Pharmacopeial Drug Library (HY-L066) was utilized in this study and obtained from MedChemExpress (NJ, United States). Additionally, Visomitin (HY-100474) was purchased from the same supplier. N-acetylcysteine amide (NAC) was purchased from Sigma (Sigma, St. Louis, MO, United States; A9165). The following primary antibodies were used for western blots: Cyclin D1 (Santa Cruz Biotechnology, Dallas, TX, United States; sc-8396), CDK4 (Santa Cruz Biotechnology; sc-23896), p16 (Cell Signaling Technology, Danvers, MA, United States; 80772S), p21 (Cell Signaling Technology; 2947S), Rb (Santa Cruz Biotechnology; sc-102), Mcl-1 (Santa Cruz Biotechnology; sc-12756), Bcl-xl (eBioscience, Vienna, Austria; 14-6994-81), Bak (Santa Cruz Biotechnology; sc-832), Bax (Santa Cruz Biotechnology; sc-7480), P-ATM (Cell Signaling Technology; 5883S), P-Histone H2A.X (Cell Signaling Technology; 9718S), SYK (Santa Cruz Biotechnology; sc-1240), phospho-SYK (Cell Signaling Technology; 2710S), and β-actin (Santa Cruz Biotechnology; sc-4778). HRP-conjugated anti-rabbit and anti-mouse secondary antibodies were obtained from Bethyl Laboratories, Inc. (A120-101p and A90-116p-33; Montgomery, TX, United States).

### 2.2 Cell lines and cell culture

The human AML cell lines HL60, U937 and mouse AML cell line M1 were purchased from Korean Cell Line Bank (Seoul, Korea). Murine bone marrow cells collected from BALB/c Slu-nu/u mice were purchased from Central Lab Animal Inc. In Seoul, Korea. These cells were cultivated in RPMI-1640 medium (Hyclone, Logan, UT, United States) supplemented with 10% fetal bovine serum (FBS; Hyclone, Melbourne, VIC, Australia), 1% L-glutamine, 1% N-2-hydroxyethylpiperazine-N′-2-ethanesulfonic acid (HEPES) buffer, and 1% penicillin/streptomycin. Cells were cultured in an incubator at 37 °C under 5% CO2.

### 2.3 Analysis of differentiation markers

Cells were seeded at a density of 3.0 × 10^5^ cells/well in 12-well plates and treated with 500 nM Visomitin (MedChemExpress, NJ, United States; HY-100474) and/or 10 mM N-acetylcysteine (NAC; Sigma-Aldrich) for 72 h. The SYK inhibitor BAY 61-3606 (Sigma) was applied at 10 µM for 72 h. After that, cells were stained with PerCP-Cy™5.5 Mouse Anti-Human CD14 antibody (BD Biosciences) for 1 h at 4 °C. Stained samples were analyzed by using flow cytometry (BD FACSAria™ Fusion Flow Cytometer, BD Biosciences, Becton Drive Franklin Lakes, NJ, United States). The result was then analyzed with the use of FlowJo 7.6 software (TreeStar).

### 2.4 Quantitative RT-PCR analysis (qRT-PCR)

Total cellular RNA was extracted using TRIzol reagent (FATRR 001; Favorgen, Wien, Austria) and transcribed into cDNA using a Prime-Script RT Reagent Kit following the manufacturer’s instructions (RR047A; Takara, Kusatsu-shi, Japan). The differentiation related genes were measured by qRT-PCR using TOPreal qPCR PreMIX SYBR Green with low ROX (RT500M; En-zynomics). PCR was performed as follows: 95 °C for 15 min, 40 cycles at 95 °C for 15 s, 59 °C for 30 s, and 72 °C for 30 s. The primer sequences used in the analysis are provided in Table 1.

**TABLE 1:** List of primers for qPCR.

Primer ID	Forward	Reverse
c-Myc	TTC​GGG​TAG​TGG​AAA​ACC​AG	CAG​CAG​CTC​GAA​TTT​CTT​CC
Myb	GGC​AGA​AAT​CGC​AAA​GCT​AC	GCA​GGG​AGT​TGA​GCT​GTA​GG
SOX4	CCA​GCA​AGA​AGG​CGA​GTT​AG	CGG​AGC​CTT​CTG​TCT​TCA​TC
JUNB	TGG​AAC​AGC​CCT​TCT​ACC​AC	GAA​GAG​GCG​AGC​TTG​AGA​GA
c/EBP-β	GAC​AAG​CAC​AGC​GAC​GAG​TA	AGC​TGC​TCC​ACC​TTC​TTC​TG
Lyz	GCC​AAA​TGG​GAG​AGT​GGT​TA	ATC​ACG​GAC​AAC​CCT​CTT​TG
c/EBP-ε	CCC​TTA​CAC​AAG​GGC​AAG​AA	CTC​TGC​CAT​GTA​CTC​CAG​CA
c-Jun	GGA​GTG​TCC​AGA​GAG​CCT​TG	GAA​AGG​CTT​GCA​AAA​GTT​CG
Egr-1	TGA​CCG​CAG​AGT​CTT​TTC​CT	TGG​GTT​GGT​CAT​GCT​CAC​TA
MMP9	TTG​ACA​GCG​ACA​AGA​AGT​GG	GCC​ATT​CAC​GTC​GTC​CTT​AT
ITGAM	AGA​ACA​ACA​TGC​CCA​GAA​CC	GCG​GTC​CCA​TAT​GAC​AGT​CT
PU.1	CCA​GCT​CAG​ATG​AGG​AGG​AG	ACC​AGT​TCC​TGT​TGG​ACC​TG
TBP	TAT​AAT​CCC​AAG​CGG​TTT​GCT​GCG	AAT​TGT​TGG​TGG​GTG​AGC​ACA​AGG
TNF-α	AGG​GAC​CTC​TCT​CTA​ATC​AG	TGG​GAG​TAG​ATG​AGG​TAC​AG
IL-1β	CCA​CAG​ACC​TTC​CAG​GAG​AAT​G	GTG​CAG​TTC​AGT​GAT​CGT​ACA​GG
IL-10	TAA​CAT​GCT​TCG​AGA​TCT​CCG​AGA	TCA​GAC​AAG​GCT​TGG​CAA​CCC​A
CD86A	CCA​TCA​GCT​TGT​CTG​TTT​CAT​TCC	GCT​GTA​ATC​CAA​GGA​ATG​TGG​TC
CD163	CAG​TGC​AGA​AAA​CCC​CAC​AA	AAA​GGA​TGA​CTG​ACG​GGA​TGA

### 2.5 Giemsa staining

Cells were seeded at a density of 2.0 × 10^5^ cells/well in 12-well plates and treated with Artemotil for 72 h. Then, cells were harvested and fixed with methanol and dried. The slides were stained with Giemsa stain (Sigma, 48,900-500ML-F, St. Louis, MO, United States). Then, cells were rinsed with deionized water, air-dried, and observed under a light microscope (Olympus Corporation, Tokyo, Japan) at 400X magnification. The stained cells were assessed for size, regularity of the cell margin, and morphological characteristics of the nuclei. Representative images were captured using Images Plus 2.0 software (Motic Co., Ltd., Xiamen, China).

### 2.6 Cell viability and proliferation

To determine cell viability, CellTiter 96 AQueous One Solution [3-(4,5-dimethylthiazol-2-yl)-5-(3-carboxymethoxyphenyl)-2] cell proliferation assays (MTS) were performed according to the manufacturer’s instructions (Promega, Madi-son, WI, United States). Cells were seeded in 96-well plate at density of 3.0 × 10^4^ cells/well and treated with indicated drugs, and cell viability was measured using MTS assay. The MTS reagent (30 μL) was added to each well and incubated at 37 °C for 4 h. Absorbance was measured at 450 nm using a GloMaxTM Microplate multi-mode reader (Promega).

The cell proliferation rates were determined using trypan blue exclusion assays. Cells were seeded at 2.0 × 10^5^ cells/well in a 24-well cell culture plate on day 0 and counted every 24 h using a hemocytometer (Marienfeld, LaudaKönigshofen, Germany) under a phase-contrast microscope (Olympus CKX41; Olympus Corporation, Tokyo, Japan) after Trypan blue staining (Gibco, 15250061).

### 2.7 Measurement of apoptosis by flow cytometry

To analyze apoptotic rates, human Acute myeloid leukemia cell lines (U937, HL60) were plated in 12-well plates at a density of 1 × 10^6^ cells per well and treated with Visomitin (500 nM) for 72 h or treated with SYK inhibitor (BAY 61-3606, 10 μM) for 24 h. The murine bone marrow cells or AML cell lines M1 were plated in 12-well plates at a density of 1 × 10^6^ cells per well and treated with Visomitin (500 nM) for 24 h. The apoptosis rate was analyzed using flow cytometry (FACSVerse, BD biosciences) after staining with FITC Annexin V Apoptosis Detection Kit I following the manufacturer’s instructions (556,547; BD biosciences, Franklin Lakes, NJ, United States). Cells were first gated based on forward and side scatter (FSC and SSC) to include only live, single cells, thereby excluding apoptotic cells from the gating process. A total of 10,000 events per singlet gate were analyzed. DMSO was used as the vehicle control for Visomitin, and the corresponding solvent was used for NAC or other drug treatments. Compensation was performed using unstained controls and single-stained samples according to the manufacturer’s instructions in the BD FACSAria™ Fusion Flow Cytometer software. The gating strategy is illustrated in Supplementary Figure S1.

### 2.8 Phagocytosis assay

U937 cells were seeded in a 12-well plate at a density of 3 × 10^5^ cells per well and treated with Visomitin at a concentration of 500 nM. After 72 h, the cells were incubated with 1 μM of FluoSpheres (F8823; Invitrogen) for 1 h at 37 °C, following the manufacturer’s instructions. The GFP-positive cell population was analyzed using flow cytometry and visualized under a light microscope at ×400 magnification.

### 2.9 Measurement of oxidative burst

Intracellular mitochondrial ROS levels were measured using Dihydrorhodamine 123 (MCE, HY101894). U937 cells were cultured at 3.0 × 10^5^ cells/mL and treated with 500 nM Visomitin for 72 h. Cells were then incubated with Dihydrorhodamine 123 at 37 °C for 30 min, and oxidative burst was assessed by flow cytometry (FACS).

### 2.10 Colony formation assay

A colony formation assay was performed as previously described. Initially, cells were seeded at a density of 1 × 10^4^ cells per well in 12-well plates and treated with Visomitin. Then, colony-forming cells were evaluated microscopically 8 days after plating. For this microscopic evaluation, a ×400 magnification was achieved using an Olympus CX31 microscope (Olympus Corporation, Tokyo, Japan). Representative images were captured using Images Plus 2.0 software (Motic Co., Ltd., Xiamen, China).

### 2.11 Western blotting

To perform Western blot, U937 cells were seeded in a 12-well plate at a density of 1 × 10^6^ per well, and treated with Visomitin and/or NAC (500 nM, 10 mM, each). Doxorubicin was treated at 100 nM. After 24 h, cells were harvested and lysed in RIPA buffer (ELPIS Biotechnology; Daejeon, Korea) with 1 mM Na-vanadate, 50 mM β-glycerophosphate disodium salt, β-mercaptoethanol (142 mM; BioWORLD, Visalia, CA, United States), ProteaseArrestTM (G-Bioscience; Maryland Heights, MO, United States), and EDTA (5 mM; G-Bioscience). Protein samples were boiled in a 5 X sample buffer at 100 °C for 10 min and loaded onto the poly-acrylamide gels. After gel electrophoresis, proteins were transferred onto Immobilon-P transfer membranes, followed by blocking in 1% bovine serum albumin (BSA; MP Bio-medicals, Santa Ana, CA, United States) dissolved in Tris-buffered saline containing 0.1% Tween-20 (TBST). Membranes were incubated with primary antibodies for 16 h at 4 °C on a rotor and washed thrice for 5 min each with TBST. The membranes were incubated with anti-mouse secondary antibodies or anti-rabbit secondary antibodies at room temperature for 1 h and rinsed thrice for 10 min each with TBST. Protein bands on membranes were treated with a chemilu-minescent substrate (EzWestLumi plus (ATTO, Osaka, Japan)) and visualized using a Luminograph II system (ATTO, Osaka, Japan) (Kim et al., 2019).

### 2.12 Analysis of caspase 3/7 activity

To evaluate caspase-3/7 enzymatic activity in AML cells, HL60 cells were treated with 500 nM Visomitin for 24 h. After treatment, Caspase-Glo 3/7 assay reagent (G8090; Promega) was added to the cells, followed by a 1-h incubation at room temperature. Luminescence was then measured using a GloMax™ Microplate multimode reader (Promega).

### 2.13 AML patient samples

Bone marrow samples from AML patients were provided by Pusan National University Hospital after obtaining informed consent and approval from the institutional review board (IRB 2403-010-137). The mutation profiles for each patient sample, patient clinical characteristics, including subtype, blast percentage, and treatment status, are summarized in Table 2, and the list of genes screened by targeted NGS is provided in Supplementary Table S2. The AML patient samples were treated with Visomitin for 24 h. Subsequently, the apoptotic rate was evaluated by trypan blue staining.

**TABLE 2:** Primary AML patient profiles.

Patient No.	Diagnosis	Source	Mutations	Blast %	Subtype	Treatment status
1	Acute monocytic leukemia	BM	IDH1 mutation, ETV6 mutation	50	M5	Naïve
2	Acute myeloid leukemia, r/o AML with maturation	BM	CEBPA mutation	52.2	M2	Naïve
3	Acute myeloid leukemia, r/o AML-MRC	BM	—	20.9	M0	Naïve
4	AML-MRC	BM	—	32.6	M0	Naïve
5	Acute myelomonocytic leukemia, suggestive	BM	TP53 mutation, NUDT15 mutation	22.5	M0	Naïve

### 2.14 Mitochondrial ROS measurement

Human AML cells were seeded in a 12-well plate at a density of 3.0 × 10^5^ cells/well, while mouse normal bone marrow cells or mouse AML cells were seeded in a 12-well plate at a density of 1.0 × 10^6^ cells/well. The cells were treated with 500 nM Visomitin for 24 h. Measurement of mitochondrial superoxide using flowcytometry (BD FACSAria™ Fusion Flow Cytometer; BD Biosciences) was carried out after MitoSOX Red (MedChemExpress; HY-D1055) staining at a final concentration of 5 μM for 30 min, at 37 °C, in dark, according to the manufacturer’s recommendations. The result was then analyzed with the use of FlowJo 7.6 software (TreeStar).

### 2.15 Mitochondrial complex I activity assay

To assess complex I activity, U937 cells (1.0 × 10^7^) were treated with 500 nM Visomitin for 24 h. Complex I activity was then measured using the Complex I Enzyme Activity Microplate Assay Kit (Colorimetric; Abcam, ab109721) according to the manufacturer’s instructions. Luminescence was measured at 450 nm using a GloMax™ Microplate Multimode Reader (Promega).

### 2.16 Genetic modulation of SYK in AML

For transient expression of SYK, U937 cells were cultured at 3.0 × 10^5^ cells/well and transfected with 100 ng of SYK overexpression vector or control vector using Metafectene PRO (Biontex, Germany) according to the manufacturer’s instructions. Cells were maintained in RPMI-1640 medium without penicillin/streptomycin. The DNA construct was kindly provided by Prof. Jae Youl Cho, Department of Bioinformatics and Life Science, Soongsil University, Korea.

### 2.17 *In vivo* analysis

All animal care and experimental procedures were approved by the Pusan National University-Institutional Animal Care and Use Committee (Approval No. PNU-2021-3056) and carried out in compliance with the university’s scientific research guidelines and regulations.

Male BALB/c Slu-nu/u mice, aged 4 weeks, were procured from Central Lab. Animal Inc. In Seoul, Korea. The mice were allocated randomly to different experimental groups.

Xenograft tumors were established by injecting 1.0 × 10^7^ U937 cells subcutaneously into the flank of each mouse, with daily monitoring of tumor growth. The mice were allocated randomly into a vehicle group and a treatment group. An Visomitin solution was prepared in DMSO and delivered via an intraperitoneal injection. Mice bearing xenograft tumors were treated with 4 mg kg^-1^ Visomitin for 1 week and 2 mg kg^-1^ Visomitin for the remaining days. The initial dose was chosen based on previously reported toxicity studies of Visomitin (Petrov et al., 2016) and *in vitro* activity, and the reduction was made after a slight body weight decrease to maintain tolerability while preserving anti-tumor efficacy. The tumor volumes (mm^3^) were calculated using the following formula: (A) × (B2)/2, where A was the largest diameter (mm) and B the smallest (mm). Cyclophosphamide and cyclosporine were administered intraperitoneally to mice for immunosuppression 7 days before tumor cell injection, at doses of 60 mg/kg and 10 mg/kg, respectively. Xenograft tumor tissues were disrupted between the frosted ends of microscope slides, and a single tumor cell suspension was produced using a cell strainer (SPL; 93,100) to remove the aggregates and debris. The collected tumor cells were washed with PBS, centrifuged for 5 minutes at 2000 rpm at room temperature, and stained with MitoSOX Red of 5 μM for 30 min, at 37 °C, in dark, according to the manufacturer’s recommendations. The stained samples were analyzed by flow cytometry, and the results were processed using FlowJo 7.6 software (TreeStar).

### 2.18 Immunohistochemical (IHC) staining

IHC staining were performed as previously described (Kim et al., 2018). For IHC staining of Ki-67 in tissues, a polyclonal anti-Ki-67 antibody and a polymer-horseradish peroxidase anti-rabbit antibody were used as a primary and a secondary antibody, respectively, followed by 3,3-diaminobenzidine treatment to visualize the proteins.

### 2.19 Hematoxylin and Eosin (H&E) staining

The tissues from mice were fixed with a 10% neutral buffered formalin solution (Sigma; St Louis, MO, United States) and embedded in paraffin. Paraffin sample blocks were sectioned into 4 M, deparaffinized, hydrated, and stained with H&E. H&E-stained samples were observed with an Olympus CX31 microscope (Olympus Corporation, Tokyo, Japan) at 100 magnification. Representative images were captured using Images Plus 2.0 software (Motic Co. Ltd., Xiamen, China) (Kim et al., 2018).

### 2.20 Statistical analysis

All procedures were replicated independently a minimum of three times to ensure the reliability of the experimental results. The data are represented as the mean ± standard deviation (SD). Either a Mann–Whitney U test or a one-way ANOVA, followed by Tukey’s post hoc analysis, were applied to identify the significant differences. These statistical analyses were performed using Microsoft Office Excel and GraphPad Prism five software (GraphPad Software, Inc., San Diego, CA, United States).

## 3 Results

### 3.1 Visomitin induces cell differentiation in AML

**FIGURE 1:**
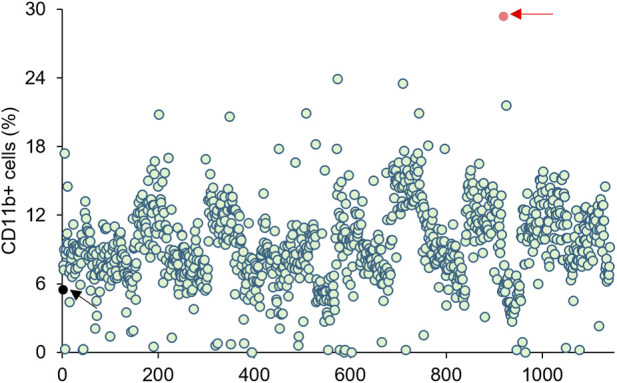
Chemical screening. CD11b expression was detected by FACS analysis in U937 cells after treatment of indicated drugs for 72 h (Black arrow, control; red arrow, Visomitin). Hits were defined based on a cutoff of chemical/ATRA ratio >0.5 (ATRA 51.4%, Visomitin 29.4%).

To identify potential differentiation-inducing agents for acute myeloid leukemia (AML), we randomly selected and screened 1,140 compounds from the FDA-Approved and Pharmacopeial Drug Library using the human AML cell line U937 (Supplementary Table S1). CD11b expression—a cell surface marker upregulated during myeloid cell differentiation and commonly used as a primary indicator—was measured by flow cytometry (FACS) analysis. Hits were defined based on a chemical/ATRA activity ratio greater than 0.5. Among the compounds, Visomitin exhibited a ratio of 0.572 and was the only compound meeting the predefined cutoff, demonstrating AML-selective activity. Visomitin also notably increased CD11b expression and was therefore prioritized as a potential differentiating agent for AML treatment (Figure 1). Other compounds did not surpass the threshold, highlighting the specificity of Visomitin as a candidate.

**FIGURE 2:**
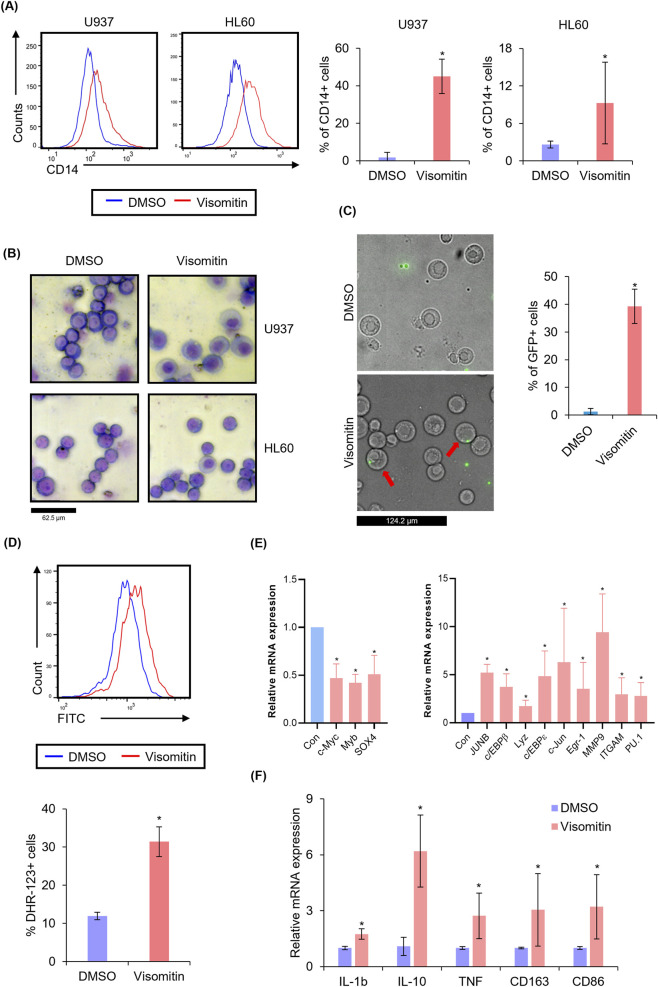
Visomitin induces cell differentiation in AML. **(A)** CD14 expression was detected by FACS analysis after the Visomitin treatment (500 nM) for 72 h in U937 and HL 60 cell lines. **(B)** Giemsa staining results showing cell differentiation in AML cell lines in response to a 500 nM Visomitin treatment for 72 h in HL60 and U937 cell lines. **(C)** Phagocytosis was assessed by flow cytometry and fluorescence microscopy in U937 cells, with representative images shown from three independent experiments. **(D)** Oxidative burst was assessed by FACS analysis in U937 cells following 72-h treatment with 500 nM Visomitin using Dihydrorhodamine-123 staining. **(E)** Relative expression levels of myeloid differentiation-related genes were measured by qRT-PCR in U937 cells at 72-h intervals post 500 nM Visomitin treatment. **(F)** Relative expression levels of macrophage gene signatures were measured by qRT-PCR in U937 cells after 72-h treatment with 500 nM Visomitin. The data are presented as mean ± SD (n = 3) for each group and statistical analysis was performed using the Student’s t-test and two-tailed Mann–Whitney U test (*p < 0.05).

Next, to further evaluate the therapeutic potential of Visomitin in AML, we assessed its ability to induce differentiation in two human AML cell lines, U937 and HL60, using CD14 as a surface marker. CD14 is known to be upregulated during the differentiation of myeloid cells into macrophages. FACS analysis revealed that Visomitin treatment significantly upregulated CD14 expression in both U937 and HL60 cells (Figure 2A). Morphological changes indicative of myeloid differentiation, such as the emergence of granulocyte-like structures with multilobed nuclei and cytoplasmic granules, were observed via Giemsa staining (Figure 2B). The phagocytic activity of cells with granulocyte-like structures induced by Visomitin treatment was assessed using FluoSphere beads and was found to be increased compared to the control group (Figure 2C). In addition, oxidative burst activity—an established functional feature of mature myeloid cells detectable by DHR-123 oxidation (Trayner et al., 1995; Smoljo et al., 2023)—was significantly increased in Visomitin-treated cells (Figure 2D), providing further evidence of functional differentiation. Together, these morphological changes, increased phagocytosis, and enhanced oxidative burst suggest that Visomitin efficiently induces differentiation in AML cells. Additionally, Visomitin treatment upregulated nine myeloid differentiation markers, including MMP9, c-Jun, and ITGAM. Conversely, it significantly reduced the expression of c-Myc, Myb, and SOX4, which are known to inhibit the differentiation of normal hematopoietic cells and to promote the onset and progression of cancer (Figure 2E). Furthermore, to assess macrophage gene signatures as an additional measure of functional differentiation, we examined mRNA expression of M1-associated markers (CD86, TNF-α, IL-1β) and M2-associated markers (CD163, IL-10) (Yuan et al., 2015; Murray, 2017; Wu et al., 2022; Strizova et al., 2023). All markers were significantly upregulated upon Visomitin treatment in U937 cells, indicating differentiation toward a macrophage-like phenotype (Figure 2F). These results collectively suggest that Visomitin induces differentiation of AML cells.

### 3.2 Visomitin induces cell cycle arrest in AML cells

**FIGURE 3:**
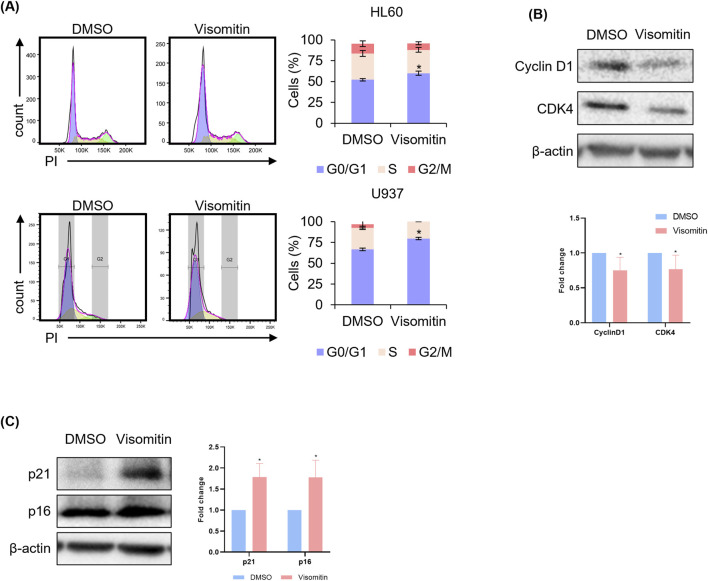
Visomitin induces cell cycle arrest in AML cells. **(A)** HL60 and U937 cells were treated with Visomitin (1 μM) for 24 h. Cell cycle was analyzed by FACS after PI staining. Expression levels of Cyclin D1 and CDK4 **(B)**, as well as p21 and p16 **(C)**, were analyzed by Western blotting after treatment with 500 nM Visomitin for 24 h in U937 cells. β-actin was used as a loading control. All data are presented as mean ± SD and representative of three independent experiments and statistical analysis was performed using the two-tailed Mann–Whitney U test (*p < 0.05).

The differentiation of AML has been reported to be closely associated with cell cycle arrest, which plays a critical role in regulating cell proliferation and differentiation (Ketley et al., 2000; Schnerch et al., 2012). In order to better understand the impact of differentiation induced by Visomitin on the cell cycle dynamics, we specifically examined the alterations in cell cycle progression and arrest in AML cell lines treated with Visomitin. Notably, a significant cell cycle arrest was observed, with an increase in the proportion of cells in the G0/G1 phase when compared to the control group following Visomitin treatment, suggesting an inhibitory effect on cell cycle progression (Figure 3A). To further investigate the underlying molecular mechanisms, Western blot analysis was performed, revealing that Visomitin treatment led to a marked downregulation in the expression levels of cyclin D1 and CDK4, both of which are essential regulators of cell cycle progression during the G0/G1 phase (Figure 3B). In addition, the expression of key cyclin dependent kinase inhibitors (CKIs), including p21 and p16, which act by binding to CDK complexes and inhibiting the activation of cyclin/CDK complexes to promote cell cycle arrest, was significantly increased following Visomitin treatment, further corroborating the observed cell cycle arrest (Figure 3C). Taken together, these findings suggest that Visomitin may have potential anti-leukemia activity by disrupting critical cell cycle regulatory pathways.

### 3.3 Visomitin suppresses cell survival by inducing apoptosis in AML cells

**FIGURE 4:**
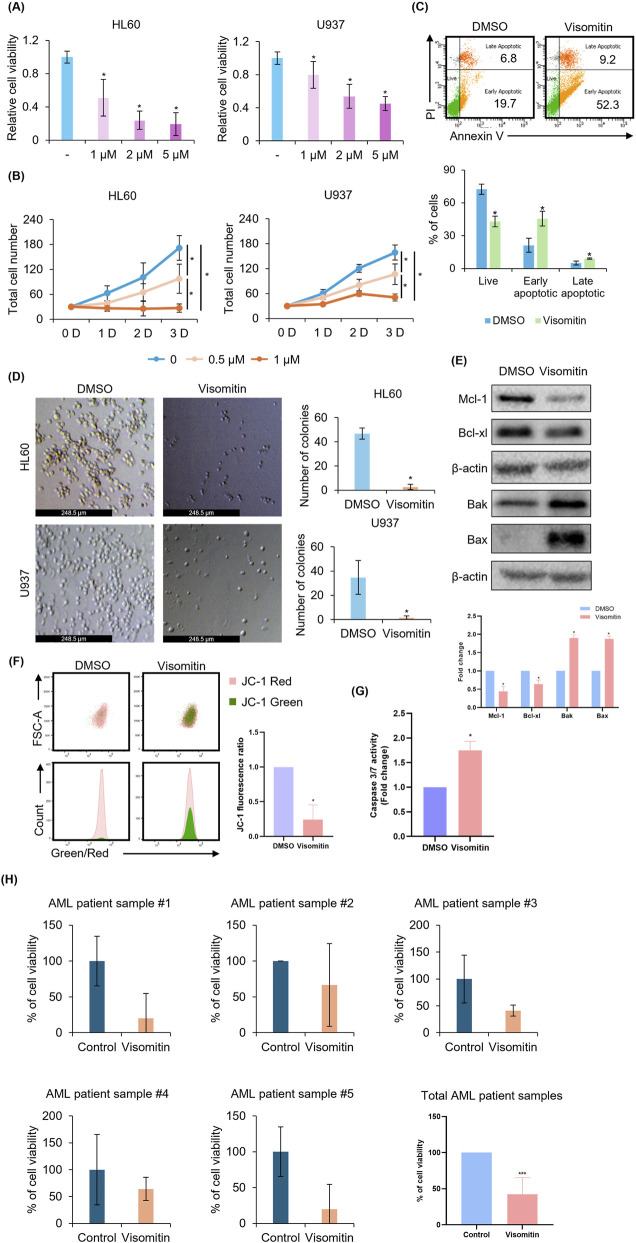
Visomitin induces cell apoptosis in AML. **(A)** HL60 and U937 cells were treated with Visomitin at indicated concentrations for 48 h. Cell viability was measured using MTS assay. **(B)** Trypan blue staining was used to count cell numbers after treatment with Visomitin in HL60 and U937 cells at the indicated times and concentrations. **(C)** The apoptotic rate was assessed by FACS after PI/Annexin V staining in U937 cells treated with 500 nM Visomitin for 72 h. Statistical analysis was performed using the one-way ANOVA (*p < 0.05). **(D)** A colony forming assay was performed and quantified in U937 and HL60 cells treated with Visomitin (500 nM) for 7 days. **(E)** Western blot analysis of Bcl-2 family members in U937 cells treated with 500 nM Visomitin. MCL-1 and Bcl-xL were analyzed after 24 h treatment; Bax and Bak after 1 h β-actin was used as a loading control. **(F)** U937 cells were treated with 500 nM Visomitin for 24 h. To monitor mitochondrial membrane potential (MMP), the cells were stained with JC-1, and the JC-1 fluorescence ratio was calculated using flow cytometry. Intact and disrupted MMP were represented by red (J-aggregates) and green (J-monomers) fluorescence, respectively. **(G)** Caspase-3/7 activity was measured by ELISA-based bioluminescence assays following 24-h treatment with 500 nM Visomitin in HL60 cells. **(H)** Trypan blue staining was used to assess cell viability after treatment with 500 nM Visomitin for 24 h in primary AML patient bone marrow cells. Statistical analysis was performed using the two-tailed Mann–Whitney U test (*p < 0.05). All data are presented as mean ± SD (n = 3) for each group.

Above data show that Visomitin significantly induced differentiation and cell cycle arrest in AML. Our next aim was to investigate whether Visomitin exhibited anti-tumor effects, particularly by inhibiting cell viability. To evaluate this, we treated U937 and HL60 cells with varying concentrations of Visomitin and assessed cell viability. The results clearly demonstrated that Visomitin treatment significantly inhibited cell viability in both cell lines in a dose-dependent manner, as shown in Figure 4A. In addition, Trypan blue staining was performed to further investigate the effect of Visomitin on cell proliferation. The staining revealed that Visomitin not only reduced cell proliferation but also exerted significant effects on cell survival (Figure 4B). Further analysis using PI and Annexin V staining followed by FACS analysis provided more insight, showing that Visomitin effectively induced apoptosis in AML cells, which is a critical mechanism contributing to its cytotoxic effects (Figure 4C). In addition, colony-forming assays were performed to assess the clonogenic potential of AML cells. These assays demonstrated that Visomitin impaired the ability of AML cells to form colonies, further confirming its inhibitory effects on cell survival (Figure 4D). Taken together, these data suggest that Visomitin exerts potent cytotoxic effects in AML.

Next, we examined the expression of Bcl-2 family proteins to better understand how Visomitin induces apoptosis in AML cells. Western blot analysis revealed changes in the expression of pro- and anti-apoptotic proteins from the Bcl-2 family after Visomitin treatment. Visomitin decreased the expression of the anti-apoptotic proteins Mcl-1 and Bcl-xL, while increasing the expression of the pro-apoptotic proteins Bax and Bak (Figure 4E). This implies that an imbalance between pro- and anti-apoptotic Bcl-2 family members may contribute to the Visomitin-induced cell death.

The misproportion disrupts the mitochondrial membrane potential (MMP), leading to the release of cytochrome c into the cytoplasm. To investigate the mitochondrial dynamics associated with the increase in Bax and Bak and the decrease in Mcl-1 and Bcl-xL, we treated AML cell lines with Visomitin and measured MMP using the mitochondrial ΔΨm-sensitive dye JC-1. Control cells with high ΔΨm displayed red-fluorescent J-aggregates formed by JC-1. However, a decrease in ΔΨm resulted in the disassembly of these aggregates into monomers, leading to a fluorescence shift from red to green. Treatment with Visomitin increased green fluorescence and significantly reduced the red/green fluorescence ratio, indicating a disruption of the MMP (Figure 4F). Additionally, we further examined caspase activation. Apoptosis is regulated by a family of cysteine-aspartic acid-specific proteases known as caspases. Upon initiation of apoptotic signaling, caspases are classified into two groups: initiator caspases (e.g., caspase-9 and -10) and effector caspases (e.g., caspase-3, -6, and -7). Visomitin was found to induce caspase-3/7 activity (Figure 4G). These results suggest that Visomitin primarily regulates Bcl-2 family proteins, thereby activating the intrinsic apoptosis pathway, destabilizing mitochondrial functions, and inducing apoptosis. Collectively, Visomitin inhibits AML cell survival by inducing differentiation, cell cycle arrest, inhibiting cell proliferation, and promoting apoptosis, ultimately leading to the anti-cancer effects in AML.

To further validate the anti-tumor effects of Visomitin in a clinical context, we treated five primary AML patient samples with Visomitin. Remarkably, treatment with Visomitin led to a reduction in cell proliferation in all patient samples, further supporting its potent cytotoxic effects in AML (Figure 4H). Although the number of samples is small and robust correlations between individual genetic alterations and drug response cannot be determined, treatment with Visomitin reduced cell viability by 80% in patient 1, 33.3% in patient 2, 58.8% in patient 3, 35.7% in patient 4, and 80% in patient 5, suggesting that its anti-leukemic activity is consistent across diverse genetic backgrounds. These results suggest that Visomitin’s ability to inhibit cell proliferation is not limited to cell lines but extends to primary AML patient cells as well, highlighting its potential therapeutic relevance in clinical settings.

### 3.4 ROS mediates visomitin-induced anti-AML effects

**FIGURE 5:**
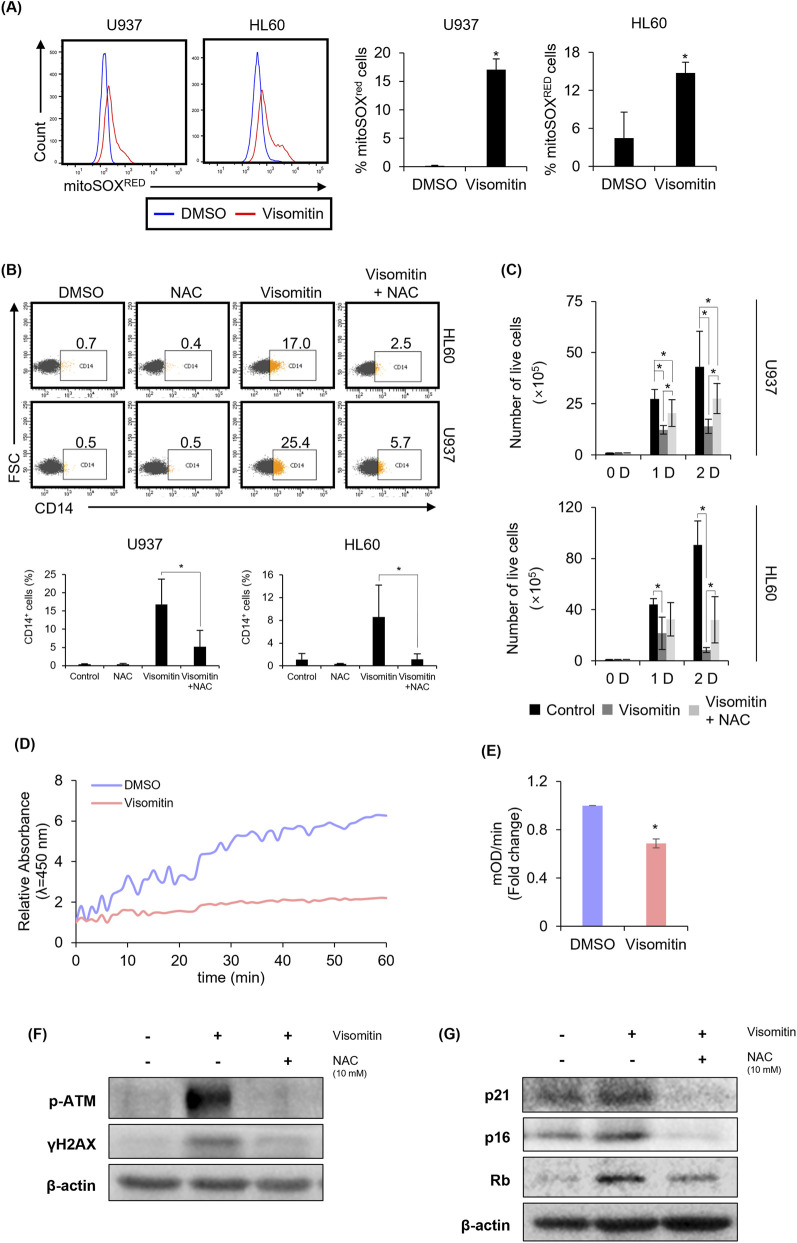
Visomitin-induced ROS accumulation causes differentiation and apoptosis in AML. **(A)** U937 and HL cells were treated with Visomitin at 500 nM for 72 h. Mitochondrial ROS levels were measured by flow cytometry after MitoSOX™ Red staining. The statistical analysis was performed using the two-tailed Mann–Whitney U test (*p < 0.05). **(B)** U937 and HL 60 cells were treated with NAC and/or Visomitin (10 mM and 500 nM, respectively) for 72 h. Flow cytometry was used to measure CD14 expression. **(C)** Trypan blue staining was used to count number of live cells in U937 and HL 60 treated with NAC and/or Visomitin for indicated time (10 mM and 500 nM, respectively). The statistical analysis was performed using the one-way ANOVA (*p < 0.05). **(D)** Relative absorbance at 450 nm over time (minutes) in U937 cells treated with 500 nM Visomitin for 24 h. Data are presented as mean ± SD from three independent experiments. **(E)** Complex I activity (mOD/min) in U937 cells after Visomitin treatment, expressed as fold change relative to control. Statistical significance was determined by Student’s t-test (p < 0.05). Expression of phospho-ATM, γH2AX **(F)**, p21, p16, and Rb **(G)** was detected by Western blotting in U937 cells after co-treatment with Visomitin, with or without NAC (500 nM and 10 mM, respectively) for 24 h. The loading control was verified using β-actin. All data are presented as mean ± SD (n = 3) for each group.

Unlike the reported potent antioxidant activity of Visomitin, as shown in Figure 5A, we found that Visomitin-treated AML cells exhibited higher mitochondrial ROS (mtROS) levels compared to the control group. We hypothesized that Visomitin contributes to apoptosis and differentiation in AML cells by accumulating mtROS, and to test this hypothesis, we assessed cell counting and CD14 expression after Visomitin treatment, both in the presence and absence of N-acetyl-L-cysteine (NAC). The results showed that NAC significantly restored cell differentiation and survival by inhibiting Visomitin-induced mtROS generation (Figures 5B,C). Next, we aimed to explore the mechanism by which Visomitin-induced mtROS accumulation leads to apoptosis and differentiation in AML cells. To investigate whether the accumulation of mtROS is linked to mitochondrial dysfunction, we performed a mitochondrial complex I activity assay. Visomitin treatment significantly reduced complex I activity, as indicated by a decreased rate of OD increase (mOD/min) compared to the control (Figures 5D,E). These results suggest that inhibition of complex I may contribute to the selective accumulation of ROS in AML cells through impaired electron transfer and increased electron leakage (Li et al., 2003; Hirst, 2013; Jang and Javadov, 2018), providing a potential mechanistic explanation for the pro-oxidant effect of Visomitin in malignant mitochondria. High levels of ROS have been reported to induce oxidative stress and damage cellular components, including lipid membranes and nucleic acids. To determine whether ROS-induced DNA damage contributes to apoptosis, we evaluated the expression of p-Ataxia-Telangiectasia Mutated (ATM) protein kinase and Phosphorylated H2A histone family member X (γH2AX), markers of DNA double-strand breaks, using Western blot analysis. The expression of p-ATM and γH2AX was significantly increased by Visomitin treatment in U937 cells and was abolished by NAC treatment (Figure 5F). To investigate whether ROS accumulation-induced DNA damage leads to cell cycle arrest and apoptosis (Feng et al., 2019; Kwon et al., 2025), we evaluated the activation of the p21/p16/Rb axis. We observed that the increased expression of p21, p16, and Rb following Visomitin treatment was inhibited by NAC treatment (Figure 5G). Therefore, these results suggest that ROS accumulated by Visomitin induce DNA damage and activate the p21/p16/Rb axis, leading to differentiation and apoptosis in AML cells.

### 3.5 Selective ROS accumulation in AML cells by visomitin

**FIGURE 6:**
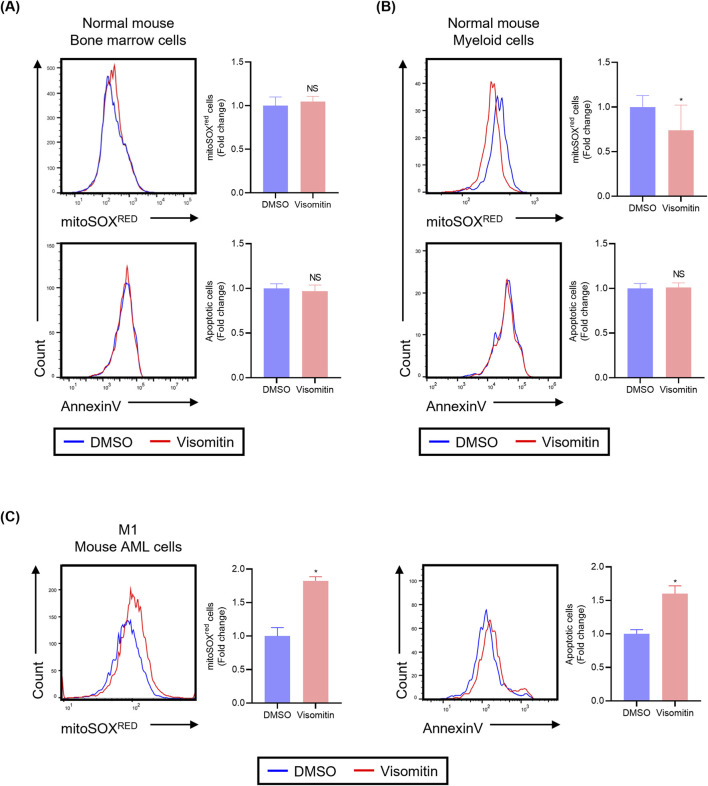
Visomitin did not increase mtROS level in normal myeloid cells. Mitochondrial ROS levels and apoptosis were assessed by flow cytometry following treatment with 500 nM Visomitin for 24 h. Analyses were performed on **(A)** normal mouse bone marrow cells, **(B)** myeloid cells (CD45^+^/CD11b^+^ double-positive cells isolated from bone marrow), and **(C)** the mouse AML cell line M1. Apoptotic cells were identified by Annexin V staining. Data are presented as mean ± SD (n = 3 per group). Statistical significance was determined using the two-tailed Mann–Whitney U test (*p < 0.05).

Visomitin is known to accumulate in mitochondria due to its positive charge and effectively scavenge mitochondrial reactive oxygen species (mtROS) through the antioxidant action of its plastoquinyl group. Intriguingly, treatment with Visomitin resulted in a significant increase in ROS levels in AML cells (Figure 5A), which was contrary to its known ROS-inhibiting effects. To investigate whether this increase in ROS accumulation is specific to AML cells or if it reflects a previously unrecognized ROS-inducing activity of Visomitin, we examined the ROS levels in both normal mouse bone marrow cells and the mouse AML cell line M1 following Visomitin treatment. In total mouse bone marrow cells, there was no significant difference in ROS levels between the control and Visomitin treatment groups (Figure 6A). However, in the myeloid cell population (CD45+/CD11b+), ROS levels were notably decreased following Visomitin treatment compared to the control group (Figure 6B), suggesting that Visomitin may inhibit ROS accumulation in normal myeloid cells. In contrast, treatment with Visomitin significantly induced ROS accumulation in the mouse AML cell line M1 compared to the control, indicating that Visomitin specifically induces ROS accumulation in AML cells (Figure 6C). To further assess the effect of Visomitin on cell survival of normal mouse myeloid cells and mouse AML cells, we evaluated apoptosis by measuring the population of Annexin V-positive cells after exposure to Visomitin. Visomitin treatment resulted in a significant increase in the Annexin V-positive cells in M1 cells, not in normal bone marrow cells or myeloid cells (Figures 6A–C). Consistent with these findings, Visomitin treatment did not induce γH2AX expression in normal mouse bone marrow cells (Supplementary Figure S2), whereas γH2AX induction was observed in AML cells (Figure 5F), indicating a differential DNA damage response between normal and malignant cells. These findings support a model in which Visomitin exerts context-dependent redox effects: it remains non-pro-oxidant in normal hematopoietic cells, while selectively disrupting mitochondrial respiratory function in AML cells, thereby exacerbating intrinsic redox imbalance and promoting ROS-mediated apoptosis.

### 3.6 ROS accumulation activates the p21/p16/Rb axis via regulating SYK activation

**FIGURE 7:**
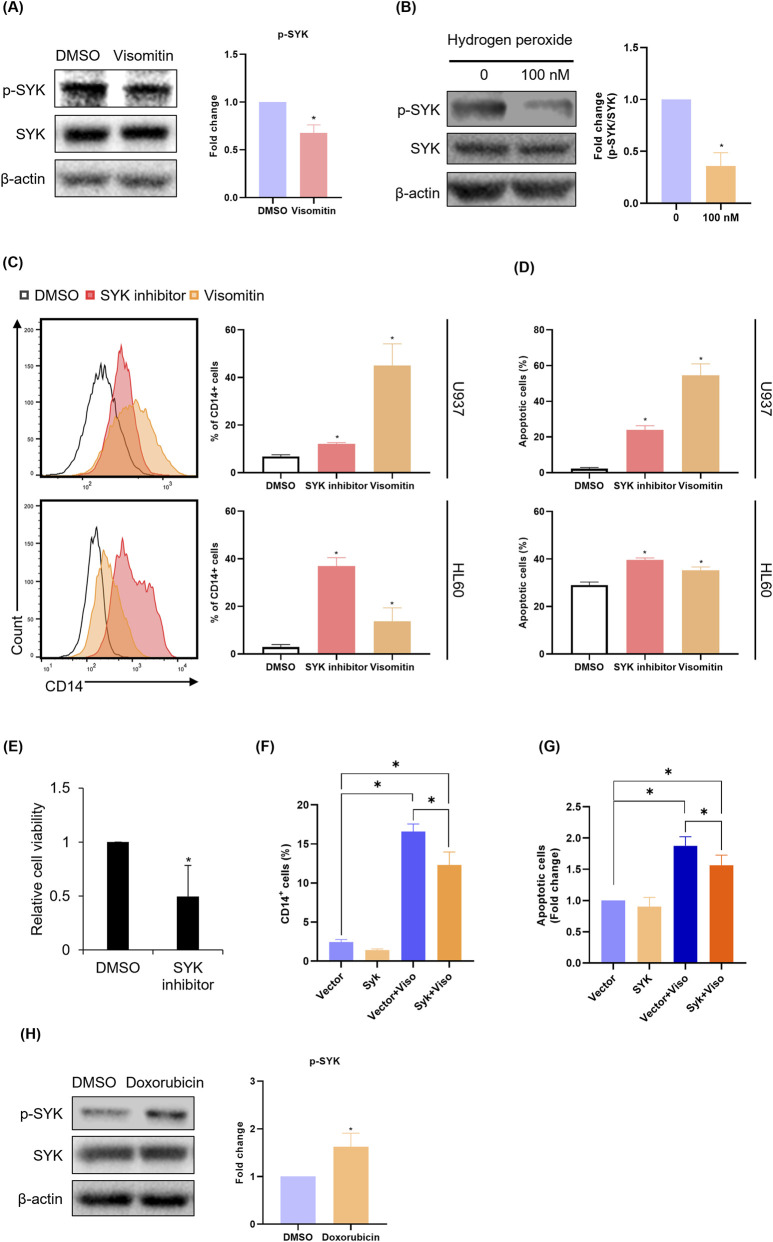
Visomitin-mediated ROS accumulation inhibits SYK activation. **(A)** U937 cells were treated with Visomitin 500 nM for 24 h. The expression levels of phospho-SYK and total SYK were detected by Western blotting. **(B)** The expression levels of phospho-SYK and total SYK were detected by Western blotting in U937 cells treated with 100 nM hydrogen peroxide for 24 h. Statistical analysis was performed using the two-tailed Mann–Whitney U test (*p < 0.05). **(C)** CD14 expression was analyzed by FACS in U937 and HL60 cells following 72 h treatment with 10 μM SYK inhibitor, BAY 61-3606. **(D)** Apoptotic cells were detected by FACS analysis after Annexin V staining in U937 and HL60 cells treated with 10 μM BAY 61-3606 for 72 h. The statistical analysis was performed using the two-tailed Mann–Whitney U test (*p < 0.05). **(E)** U937 cells were treated with 2 μM BAY 61-3606 for 24 h, and cell viability was assessed using the MTS assay. **(F)** CD14 expression was analyzed by FACS in U937 cells following 72 h treatment with 500 nM Visomitin and transfection with 100 ng of SYK overexpression vector or control vector. **(G)** Apoptotic cells were detected by FACS after Annexin V staining. Statistical analysis was performed using one-way ANOVA (*p < 0.05). **(H)** Total SYK and phospho-SYK expression were detected by Western blotting in U937 cells treated with 100 nM Doxorubicin for 24 h β-actin was used as a loading control. Statistical analysis was performed using the two-tailed Mann–Whitney U test (*p < 0.05). All data are presented as mean ± SD (n = 3) for each group.

Next, we aimed to identify the signaling pathway responsible for mediating the activation of the p16/p21/Rb axis induced by ROS accumulation. We hypothesized that the SYK could be a plausible candidate (Cheng et al., 2011; Wang et al., 2014; Qiu et al., 2015; Ishikawa et al., 2018). Treatment with Visomitin significantly reduced the expression of phospho-SYK (Figure 7A). To determine whether the blockade of the SYK activation by Visomitin was induced by ROS, we observed the phosphor-SYK after hydrogen peroxide treatment. In the group where ROS production was induced by hydrogen peroxide, the p-SYK/SYK ratio significantly decreased compared to the control (Figure 7B). To clarify whether SYK inhibition leads to cell differentiation, we treated the AML cells with the SYK inhibitor BAY 61-3606 and observed the expression of CD14. SYK inhibition significantly increased the expression of CD14 (Figure 7C). Additionally, to investigate the effect of SYK inhibition on apoptosis, we performed Annexin V staining followed by FACS analysis. Treatment with the SYK inhibitor significantly increased the proportion of apoptotic cells compared to control (Figure 7D). Similarly, SYK inhibition in AML cells reduced cell viability, further supporting the role of SYK in regulating apoptosis in AML cells (Figure 7E). To investigate the causal relationship between ROS accumulation and SYK inhibition, we performed rescue experiments by overexpressing SYK. SYK overexpression significantly rescued Visomitin-induced CD14 expression and apoptosis in AML cells (Figures 7F,G). To further explore the specificity of SYK regulation by Visomitin-induced ROS, we compared the effects of doxorubicin, a well-known ROS-inducing chemotherapeutic agent, on SYK activation (Chen et al., 2020). Notably, doxorubicin treatment led to increased SYK phosphorylation in U937 cells, consistent with previous reports linking doxorubicin-induced ROS to SYK activation (Ma et al., 2020) (Figure 7H). In contrast, Visomitin treatment resulted in SYK inactivation despite also elevating ROS levels (Figure 7A). These opposing outcomes suggest that SYK inhibition may be a Visomitin-specific effect, rather than a general consequence of ROS accumulation. Taken together, these findings suggest that ROS accumulation induced by Visomitin regulates the SYK activation, promoting both differentiation and apoptosis in AML cells.

### 3.7 Anticancer effect of visomitin in immunocompromised mice

**FIGURE 8:**
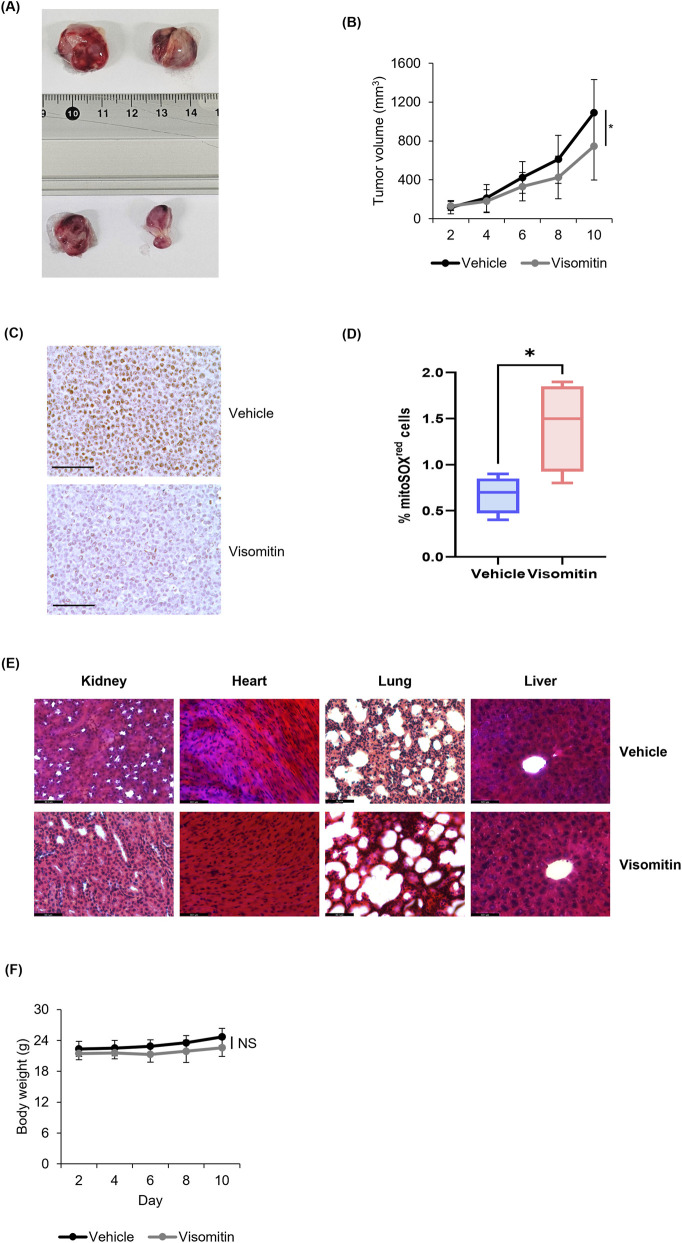
*In Vivo* Effects of Visomitin on Xenograft Tumor Growth. Representative tumor images **(A)** and quantitative analysis of tumor volume **(B)** from mice treated with vehicle or Visomitin. **(C)** Mouse xenograft tumor tissues were collected after indicated treatments and processed for Ki-67 immunohistochemistry (IHC). Ki-67-positive cells were quantified and expressed relative to the vehicle control. Scale bar, 100 μm. **(D)** Mitochondrial ROS (mtROS) levels in tumors from vehicle- or Visomitin-treated mice were measured by FACS analysis. **(E)** Representative H&E staining of major organs from vehicle- or Visomitin-treated mice. **(F)** Mouse body weight was measured every 2 days over a 10-day treatment period. The data are presented as mean ± SD (n = 3) for each group and statistical analysis was performed using the two-tailed Mann–Whitney U test (*p < 0.05).

Visomitin exerted anti-tumor effects by inducing differentiation and apoptosis *in vitro*. To investigate whether Visomitin’s anti-cancer effects would be recapitulated *in vivo*, immunocompromised athymic nude mice were inoculated with U937 AML cells, followed by administration of Visomitin via intraperitoneal injection for 10 days and evaluation of its effects on tumor growth. Although SkQ1 is primarily developed as an ophthalmic solution, previous studies have shown that it is well-tolerated in rodents even at high oral doses (up to 100 mg/kg) (Petrov et al., 2016). While precise pharmacokinetic parameters in mice are not fully reported, SkQ1 selectively accumulates in mitochondria and distributes broadly in tissues due to its lipophilic cation structure (Saric et al., 2016). Considering that *in vitro* activity was observed at ∼500 nM, we selected a higher systemic dose of 4 mg/kg to achieve sufficient exposure in the xenografted tumors. Visomitin-treated mice showed markedly reduced tumor sizes and volumes compared to the control mice (Figures 8A,B). To further evaluate the mechanism of tumor growth inhibition, Ki-67 immunohistochemistry (IHC) was performed on tumor tissues. In vehicle-treated tumors, numerous nuclei were positively stained for Ki-67, indicating active proliferation (Sun and Kaufman, 2018), whereas Visomitin-treated tumors exhibited almost no Ki-67-positive nuclei (Figure 8C), suggesting that Visomitin directly suppresses tumor cell proliferation rather than causing a general growth delay. Importantly, when the ROS levels in tumor cells from the Visomitin-treated group were measured, we observed a significant increase in intracellular ROS accumulation compared to the control, consistent with the data indicating that visomitin increased ROS levels in AML cells (Figures 5A, 8D). Evaluation of Visomitin’s cytotoxicity was conducted through H&E staining of kidney, heart, lung, and liver tissues. No tissue damage induced by Visomitin was observed in the major organs (Figure 8E). In addition, the body weights were not different between the Visomitin-treated group and the control group, which indicates that Visomitin does not systemic cytotoxicity (Figure 8F). Taken together, these results clearly show that Visomitin exhibits anti-AML effects *in vivo* by inducing ROS production.

## 4 Discussion

This study provides new insight into the potential of Visomitin as a therapeutic agent in acute myeloid leukemia (AML). We demonstrated that Visomitin not only inhibits proliferation of AML cells but also promotes their differentiation and apoptosis through mitochondrial and redox-related mechanisms. These findings extend the biological role of Visomitin beyond its established antioxidant activity and reveal a context-dependent function that may be exploited for cancer therapy.

Although Visomitin is primarily recognized as a mitochondrial-targeted antioxidant, our results show that it behaves differently in leukemic cells compared with normal hematopoietic cells. In normal myeloid cells, Visomitin reduced reactive oxygen species (ROS) levels as expected, without causing any detectable cytotoxicity or apoptosis. In contrast, AML cells exposed to Visomitin displayed a robust increase in ROS, leading to DNA damage and subsequent activation of differentiation and apoptotic pathways. This paradoxical behavior highlights a possible redox vulnerability unique to AML cells, which Visomitin may selectively target. Our complex I activity results suggest that inhibition of mitochondrial complex I may contribute to this selective ROS accumulation, providing a biologically plausible mechanism by which Visomitin, although an antioxidant, increases ROS specifically in malignant mitochondria. Such cell type–specific modulation of oxidative stress suggests a therapeutic window where leukemic cells are driven toward differentiation and death while sparing normal cells.

Mechanistically, Visomitin treatment was associated with a loss of mitochondrial membrane potential and altered expression of Bcl-2 family proteins (Cory and Adams, 2002). Specifically, the downregulation of anti-apoptotic members such as Mcl-1 and Bcl-XL, and the concomitant upregulation of pro-apoptotic proteins Bak and Bax, are consistent with activation of the intrinsic apoptotic pathway (Wei et al., 2001). These results align with previous findings that mitochondrial integrity plays a central role in chemotherapy response and resistance in AML (Choi et al., 2016; Kotschy et al., 2016; Campbell et al., 2018). The clinical success of venetoclax, a BH3-mimetic Bcl-2 inhibitor, has underscored the therapeutic relevance of targeting the mitochondrial apoptosis pathway (Guerra et al., 2019). In this context, Visomitin’s capacity to modulate the Bcl-2 network through redox signaling offers a potentially complementary or synergistic mechanism to existing treatments.

Another important observation from this study is that Visomitin promotes differentiation of AML cells, as indicated by the upregulation of CD14 and macrophage-like morphology. AML is characterized by an early block in differentiation, which contributes to uncontrolled proliferation and resistance to apoptosis. Agents that relieve this differentiation arrest have long been of interest—exemplified by all-trans retinoic acid (ATRA) therapy in acute promyelocytic leukemia (APL). Our findings suggest that Visomitin might exert a similar effect in non-APL AML subtypes by reactivating differentiation programs that are otherwise suppressed. This effect was observed even in primary AML samples carrying adverse genetic mutations such as *CEBPA* and *TP53* (Fröhling et al., 2004; Bohlander, 2005; DiNardo et al., 2015; Wong et al., 2015), both of which are often associated with defective myeloid maturation and poor clinical outcomes. The ability of Visomitin to induce differentiation in these high-risk samples suggests that its mechanism of action may transcend specific genetic contexts, targeting more fundamental cellular processes such as mitochondrial redox balance and SYK signaling.

SYK, a non-receptor tyrosine kinase, is known to regulate multiple pathways involved in hematopoietic cell proliferation and survival (Mócsai et al., 2010). Its activation has been linked to leukemogenesis through the PI3K/AKT and MAPK cascades (Hatton et al., 2012; Muro et al., 2018), and its inhibition has shown therapeutic potential in B-cell malignancies (Friedberg et al., 2010; Chen et al., 2013; Baudot et al., 2009; Buchner et al., 2009). Interestingly, our study found that Visomitin-induced ROS accumulation led to the suppression of SYK activation and the upregulation of p21/p16/Rb (Qiu et al., 2015), which are key mediators of cell cycle arrest and differentiation. This finding provides a mechanistic link between oxidative stress and the regulation of oncogenic signaling in AML. Moreover, the observation that both Visomitin and the ROS inducer hydrogen peroxide inhibited SYK activity supports the idea that oxidative modification of signaling components could underlie this response.

The dual ability of Visomitin to promote both differentiation and apoptosis is particularly attractive from a therapeutic standpoint. Conventional AML chemotherapies such as cytarabine and anthracyclines effectively reduce leukemic burden but lack selectivity, often damaging normal hematopoietic and immune cells (Jeon et al., 2023). The resulting neutropenia and immunosuppression frequently lead to infectious complications, which remain major causes of mortality. In contrast, Visomitin’s selective cytotoxicity toward AML cells—coupled with its protective antioxidant effects in normal cells—could mitigate these adverse effects while maintaining anti-leukemic efficacy. By leveraging intrinsic differences in redox homeostasis between normal and malignant cells, Visomitin may offer a novel approach to AML therapy that combines differentiation induction with selective cell death.

Despite these encouraging findings, this study has several limitations. While Visomitin reduced cell viability in primary AML samples carrying diverse genetic mutations, including CEBPA and IDH1, the number of AML cell lines and primary patient samples analyzed was limited. However, given the small sample size, definitive correlations between specific mutations and drug response could not be established, and this limited sampling may not fully capture the genetic and phenotypic heterogeneity of AML. Further studies using a broader panel of AML cell lines, patient-derived primary cells, or *in vivo* models are warranted to evaluate the variability in therapeutic response and to identify potential biomarkers predictive of sensitivity. Notably, we examined the mouse system and observed that Visomitin increased ROS levels and apoptotic cells in a mouse AML cell line (M1), while no significant changes were observed in normal mouse bone marrow or myeloid cells. However, it should be acknowledged that part of our analysis was based only on human AML cells or comparisons between human and mouse cells, which represents a limitation of the study. Thus, further validation using primary human normal hematopoietic cells would be ideal to fully account for potential species-specific differences. Additionally, while we demonstrated that ROS accumulation and SYK inhibition are associated with differentiation and apoptosis, the precise molecular events connecting these processes remain to be elucidated. Furthermore, although our *in vivo* experiments using cell line-derived xenograft models showed significant tumor inhibition, it should be noted that this model is not fully representative of AML, as it does not recapitulate bone marrow infiltration, circulating blasts, or leukemia-specific drug responses. Specifically, U937 subcutaneous xenograft models grow as solid tumors and therefore cannot model key features of hematologic malignancies, including interactions with the hematopoietic microenvironment. Nevertheless, subcutaneous xenograft models have been widely used in AML research as a practical and reproducible system for the initial evaluation of *in vivo* anti-leukemic drug efficacy, particularly for assessing tumor growth inhibition and treatment response in a controlled setting (Ranganathan et al., 2012; Ranganathan et al., 2016; Josa-Culleré et al., 2021; Qiao et al., 2021; San José-Enériz et al., 2024; George et al., 2025). In the present study, this model was employed to provide proof-of-concept evidence for the *in vivo* activity of Visomitin. Moreover, the number of animals per cohort and the limited number of experimental replicates further constrain the generalizability of these findings. Genetic AML models were not included in this study, which limits the assessment of Visomitin’s efficacy across different genetic backgrounds. Therefore, caution is needed when interpreting therapeutic efficacy, and validation in human patient-derived xenograft (PDX) models, which more closely mimic the clinical complexity of AML, will be important. Finally, the *in vivo* experiments were relatively short-term; although Visomitin treatment inhibited tumor growth in xenograft models, longer-term studies are needed to assess its systemic safety, pharmacokinetics, potential off-target effects, long-term signaling changes and the potential development of resistance.

Future work should therefore focus on several aspects. First, the selective mechanism by which Visomitin induces ROS in AML but not in normal cells should be dissected using advanced metabolomic and proteomic approaches. Second, combining Visomitin with established AML therapeutics, such as venetoclax or azacitidine, could reveal potential synergistic effects. Finally, expanding the study to larger cohorts of patient-derived xenograft models will be critical for validating its clinical relevance and determining which AML subtypes may benefit most from this treatment.

**FIGURE 9:**
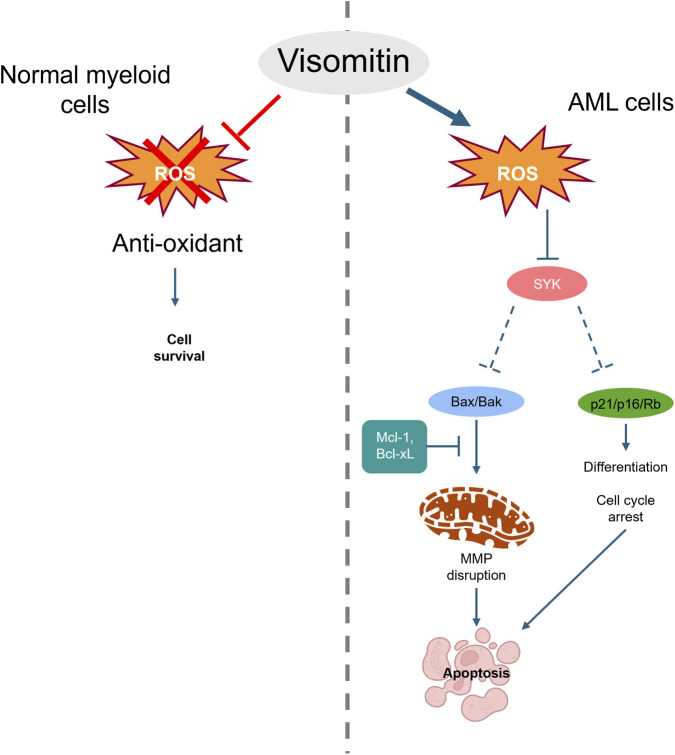
Diagram summarizing the key findings of this study. This figure presents the proposed molecular mechanisms by which Visomitin exerts its effects in AML, highlighting its potential modulation of the ROS-dependent SYK inactivation that leads to cell differentiation and apoptosis. It offers an integrated view of the hypothesized intracellular events relevant to AML therapy.

In conclusion, this study highlights the potential of Visomitin as a novel therapeutic candidate for the treatment of acute myeloid leukemia (AML). We showed that Visomitin induces differentiation and promotes apoptosis in AML cells, potentially through ROS accumulation and disruption of mitochondrial function. Our findings also suggest that Visomitin may influence the apoptotic pathway by modulating Bcl-2 family proteins and contributing to ROS-dependent SYK inactivation. These effects may help address key challenges in AML, such as chemotherapy resistance and impaired differentiation. Overall, this work provides a foundation for future studies aimed at developing targeted therapeutic strategies based on the modulation of redox and apoptotic pathways in AML (Figure 9).

## Data Availability

The original contributions presented in the study are included in the article/Supplementary Material, further inquiries can be directed to the corresponding authors.
