# Supplementary material for: Visomitin as a differentiation-inducing therapeutic agent through SYK inhibition in AML
Source: Front Pharmacol. 2026 Feb 24;17:1741351. doi: 10.3389/fphar.2026.1741351 (PMC12971925; doi:10.3389/fphar.2026.1741351)
Supplement: Supplementary file 1 [file Table2.pdf]

Supplementary data

Supplementary Table 2.

List of genes screened by targeted NGS

Test Name	Genes	
	Required genes	Optional genes
NGS-Acute Myeloid Leukemia panel	CEBPA, FLT3, IDH1, IDH2, JAK2, KIT, NPM1, RUNX1, TP53	ABL1, ACD, AFF3, AKT1, ALK, ANK1, ANKRD26, ARID1A, ASXL1, ATG2B, ATM, ATRX, B2M, BCL2, BCL6, BCOR, BCORL1, BCLR, BIRC3, BLM, BRAF, BRCA1, BRCA2, BRIP1, BTK, CALR, CBL, CCND1, CD58, CD79B, CDKN2A, CDKN2B, CFH, CREBBP, CRLF2, CSF1R, CSF3R, CTC1, CTCF, CXCR4, DIS3, DKC1, DNM2, DNMT3A, DUSP22, ECT2L, EFL1, EGFR, EGR1, ELANE, EP300, EPB42, EPCAM, EPOR, ERCC4, ETNK1, ETV6, EZH2, F2, F5, F7, F8, FAM46C, FANCA, FANCB, FANCC, FANCD2, FANCE, FANCF, FANCG, FANCI, FANCL, FANCM, FBXW7, FGFR1, FGFR3, G6PD, GATA1, GATA2, GATA3, GNAS, GP1BA, GP1BB, GP8, GSKIP, HBA1, HBA2, HBB, HCLS1, HPS1, ID3, IGF1R, IKZF1, IL2RG, IL7R, IRF4, ITGA2B, ITGB3, JAK1, JAK3, KMT2A, KMT2C, KMT2D, KRAS, LAPTM5, LRP1B, MAP2K1, MAP2K2, MEF2B, MLH1, MPL, MSH2, MSH6, MYC, MYD88, NF1, NF2, NHP2, NOP10, NOTCH1, NOTCH2, NRAS, NSD2, NUDT15, PALB2, PARN, PAX5, PBX1, PDGFRA, PDGFRB, PHF6, PIGA, PLG, PMS2, PRDM1, PRF1, PROC, PROS1, PSMG2, PTEN, PTPN11, RAD51, RAD51C, RAF1, RB1, RBBP6, RELN, RPL5, RPS19, RTEL1, SAMD9, SAMD9L, SBDS, SERPINC1, SETBP1, SF3B1, SH2B3, SHOC2, SLC4A1, SLX4, SOS1, SPRED1, SPTA1, SPTB, SRP71, SRSF2, STAG2, STAT2, STAT3, STAT5B, SUZ12, TAL1, TCF3, TERC, TERT, TET2, TGF,BR2, TINF2, TNFAIP3, TNFRSF14, TPMT, TRAF3, TYK2, U2AF1, UNC13D, VWF, WAS, WRAP53, WT1, XBP1, ZRSR2
