# Supplementary material for: Visomitin as a differentiation-inducing therapeutic agent through SYK inhibition in AML
Source: Front Pharmacol. 2026 Feb 24;17:1741351. doi: 10.3389/fphar.2026.1741351 (PMC12971925; doi:10.3389/fphar.2026.1741351)
Supplement: Supplementary file 2 [file Image2.pdf]

Supplementary Figure 2.

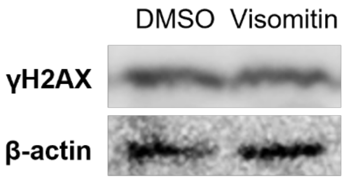

Supplementary Figure 2. Visomitin did not induce a DNA damage response in normal mouse bone marrow cells. γH2AX expression was analyzed by Western blotting in normal mouse bone marrow cells after treatment with 500 nM Visomitin for 24 h. β-actin was used as a loading control. Data are representative of three independent experiments.
