# Supplementary material for: Visomitin as a differentiation-inducing therapeutic agent through SYK inhibition in AML
Source: Front Pharmacol. 2026 Feb 24;17:1741351. doi: 10.3389/fphar.2026.1741351 (PMC12971925; doi:10.3389/fphar.2026.1741351)
Supplement: Supplementary file 4 [file Image1.pdf]

Supplementary Figure 1.

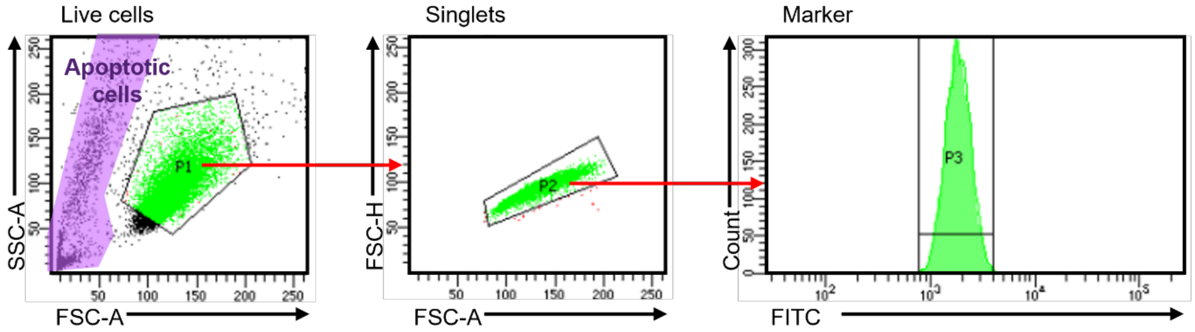

Supplementary Figure 1. FSC/SSC plot of AML cells with gating for live cells based on the size and granularity. The green dot indicates cells included in the live cell gate. In the live cell gate, gating was performed diagonally for single cells in the FSC-A vs. FSC-H plot. The singlet population confirmed the expression of the markers.
